# Multiple oligomeric structures of a bacterial small heat shock protein

**DOI:** 10.1038/srep24019

**Published:** 2016-04-07

**Authors:** Nandini Mani, Spraha Bhandari, Rodolfo Moreno, Liya Hu, B. V. Venkataram Prasad, Kaza Suguna

**Affiliations:** 1 Molecular Biophysics Unit, Indian Institute of Science, Bangalore, India; 2 Verna and Marrs McLean Department of Biochemistry and Molecular Biology, Baylor College of Medicine, Houston, United States

## Abstract

Small heat shock proteins are ubiquitous molecular chaperones that form the first line of defence against the detrimental effects of cellular stress. Under conditions of stress they undergo drastic conformational rearrangements in order to bind to misfolded substrate proteins and prevent cellular protein aggregation. Owing to the dynamic nature of small heat shock protein oligomers, elucidating the structural basis of chaperone action and oligomerization still remains a challenge. In order to understand the organization of sHSP oligomers, we have determined crystal structures of a small heat shock protein from *Salmonella typhimurium* in a dimeric form and two higher oligomeric forms: an 18-mer and a 24-mer. Though the core dimer structure is conserved in all the forms, structural heterogeneity arises due to variation in the terminal regions.

Small Heat Shock Proteins (sHSPs) are ATP-independent chaperones which prevent irreversible protein aggregation by binding to misfolded substrate proteins. Their drastic upregulation during stress, combined with their propensity to bind non-native states of substrate proteins renders them the first line of defence against the deleterious effects of cellular stress^1^. sHSPs thus play pivotal roles in modulating key cellular processes such as signal transduction, apoptosis, host-pathogen interactions and inhibition of protein aggregation and fibrillation^2^. Most sHSPs exist as large oligomers which undergo drastic rearrangement or dissociation into dimers upon activation during stress, in order to execute their chaperone function. Mechanistic insight into this conformational change and subsequent substrate binding is lacking, primarily due to the limited number of oligomeric structures determined. Amongst the different protein families, sHSPs probably exhibit the most diversity in oligomerization. Oligomeric assemblies vary not only across different species, but also within sHSPs of the same organism. Many sHSPs have been shown to co-exist in different oligomeric states in solution, either in dynamic equilibrium or upon varying experimental conditions^1^. Crystal structures of three different oligomeric assemblies have been reported so far: Octahedral 24-mers of *Methanococcus jannaschii* Hsp16.5 and *Sulfolobus tokodaii* Hsp14, double-disc dodecamer of wheat Hsp16.9, and ellipsoidal 16-mer of *Schizosaccharomyces pombe* Hsp16. Two other oligomeric assemblies have been reported from electron microscopy studies: tetrahedral dodecamer of *Mycobacterium tuberculosis* Hsp16.3 and cubic 24-mer of yeast Hsp26^1^. Monomers from all five distinct oligomeric assemblies contain a central, conserved α-crystallin domain (ACD) which adopts a β-sandwich fold, flanked by highly variable N- and C-terminal regions. In non-metazoans, the basic building unit is a symmetric dimer formed by strand exchange in the ACD. Though the mode of dimerization is different in metazoans and non-metazoans, in all archaeal, bacterial and eukaryotic high-resolution sHSP structures reported till date, a conserved arginine from one ACD forms an inter-subunit ionic interaction with a conserved aspartate or glutamate^3^. In all oligomeric structures, an IXI motif in the C-terminus binds in a hydrophobic groove on the ACD of a neighbouring dimer, to facilitate the formation of higher-order oligomers^4^,^5^,^6^. The ordered N-terminal arms of wheat Hsp16.9 and *S. pombe* Hsp16 also participate in inter-dimer interactions. However, the lack of defined electron density for the N-terminal regions in other structures points to its flexible nature and has hindered the understanding of its role in oligomerization. Despite the presence of the conserved motifs described above, sHSPs can adopt a wide range of quaternary structures and exist in solution as an ensemble of different oligomers^1^. The underlying dynamic nature of sHSP oligomers is highlighted by recent findings that different oligomeric forms of human HspB1 may interact with different substrates, and that the dodecamer of the wheat Hsp16.9 is likely to adopt several structural assemblies^7^.

Aggregation Suppressing Protein A (AgsA), an sHSP from the pathogenic bacterium *Salmonella enterica* has been reported to form dynamic oligomers^8^*. S. enterica* serovars *typhi* and *typhimurium* express three sHSPs: IbpA, IbpB and AgsA^9^. At temperatures higher than 42 °C, the most strongly induced sHSP in *S. typhimurium* is AgsA. Unlike most other sHSPs, at high temperatures AgsA forms fibrils, the length and thickness of which increase with temperature^8^. Further, the AgsA sequence lacks the highly conserved arginine and the aspartate/glutamate which mediate a key intra-dimer interaction (Fig. 1). An earlier study on AgsA from *S. typhimurium* has demonstrated the *in vivo* chaperone activity of the wild type protein and several of its truncation mutants. When deletions were confined to the first 23 residues on the N-terminus and the last 11 residues on the C-terminus, the protein was found to be active *in vivo*^10^. Investigations *in vitro* have revealed that both the chaperone activity and oligomerization of AgsA vary drastically with small changes in the lengths of the termini that flank the ACD^10^. Different truncation constructs adopt diverse oligomeric states, ranging from dimers, trimers and tetramers to 15-, 21- and 22-mers. Constructs that cannot form oligomers higher than dimers are deficient in chaperone activity towards highly denatured substrates. Further experiments revealed that an equilibrium between different oligomeric forms of AgsA is essential for its chaperone activity^8^,^10^. In order to enhance our understanding of sHSP oligomerization and to probe the roles played by both termini in mediating oligomer assembly, we undertook the structural characterization of AgsA.

## Results

**Figure 1:**
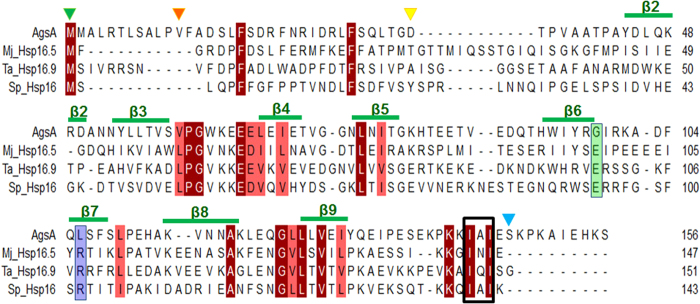
Structure-based sequence alignment of AgsA, *M. jannaschii* Hsp16.5 (Mj_Hsp16.5), wheat Hsp16.9 (Ta_Hsp16.9) and *S. pombe* Hsp16 (Sp_Hsp16). Identical residues are indicated in red, similar residues in pink. Conserved arginine is highlighted in lavender, glutamate in green, IXI motif shown in box. Arrowheads indicate N-terminus of AgsA:DelC (green), AgsA:DD (orange), degraded fragment of AgsA:His-DD (yellow). Blue arrowhead indicates C-termini of all three. Secondary structure elements of AgsA:His-DD are indicated above alignment.

Along with the wild type protein, we cloned different truncation constructs: AgsA:DelC lacking 9 residues at the C-terminus, double deletion AgsA:DD lacking 11 residues at the N-terminus and 9 residues at the C-terminus, and AgsA:His-DD, a hexa-histidine tagged double deletion construct (Fig. 1). These truncations do not extend into the amino acid range defined to be indispensable for chaperone activity^10^. We investigated the multimeric nature exhibited by AgsA and its mutants in solution and in crystalline state, and also studied their chaperone activity by lysozyme aggregation assay.

### Oligomeric states of different AgsA constructs

**Figure 2:**
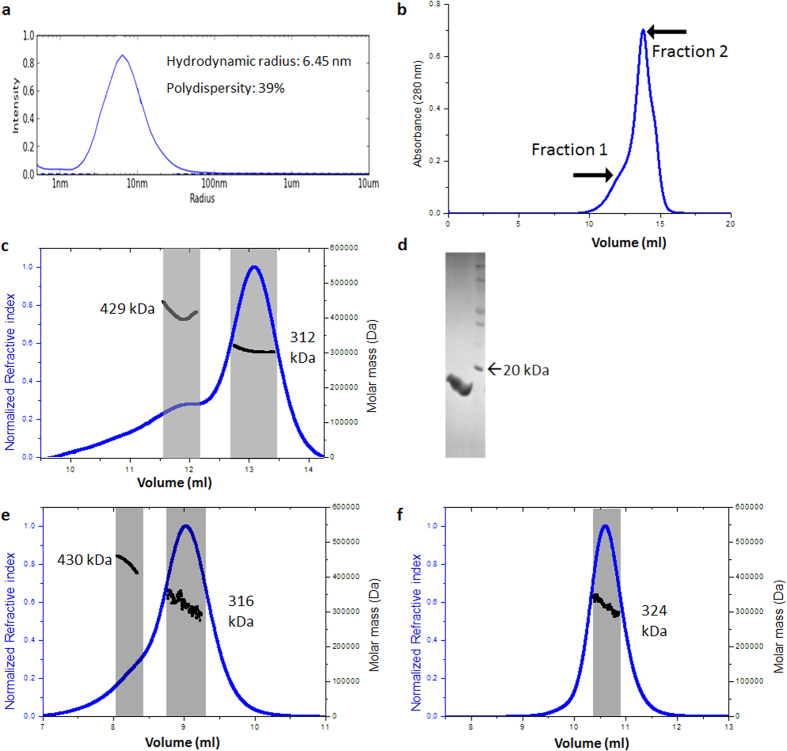
Solution studies of AgsA wild type (**a**) Radius distribution from dynamic light scattering. (**b**) Size exclusion profile. Fractions collected separately have been marked as fraction 1 and fraction 2. (**c**) SEC-MALS profile with peaks highlighted in grey for unseparated wild type protein, (**d**) SDS-PAGE profile of intact injected protein, with molecular weight marker (**e**) SEC-MALS profile of separated fraction 1 and (**f**) separated fraction 2. Expected mass for 18-mer: 318 kDa, 24-mer: 424 kDa.

In solution, the wild type protein exhibited polydispersity, as demonstrated by the dynamic light scattering and gel filtration profiles (Fig. 2a,b). Experiments with size exclusion chromatography coupled to multi-angle light scattering (SEC-MALS) revealed that the wild type protein was composed of two oligomers: A major fraction (78%) of 18-mers and a minor fraction (22%) of 24-mers (Fig. 2c,d). These two fractions, obtained after size exclusion chromatography, were separately subjected to SEC-MALS analysis. The 18-mer which is a major species retained its oligomeric state whereas the 24-mer further dissociated into a major fraction of 18-mers (87%) and a minor fraction of 24-mers (13%) (Fig. 2e,f).

**Figure 3:**
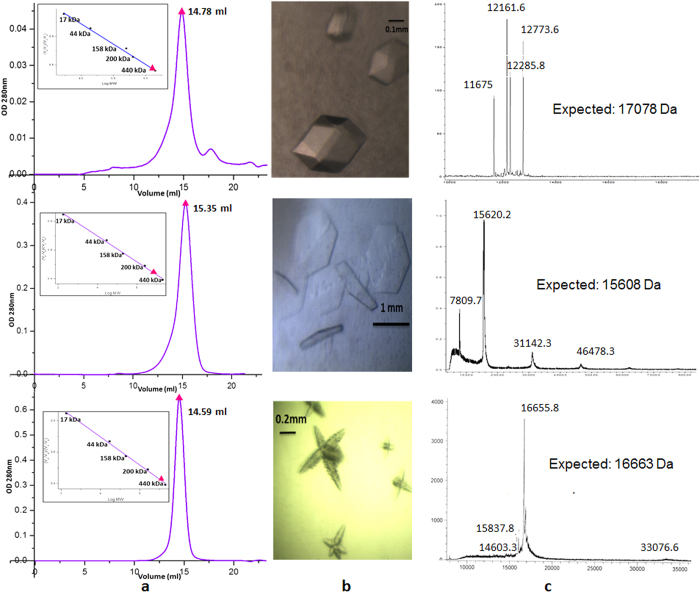
For AgsA:HisDD (top panel), AgsA:DD (middle panel) and AgsA:DelC (bottom panel): (**a**) Size exclusion chromatography with inset of standard curve (**b**) Picture of crystal (**c**) MALDI/TOF spectrum of crystal.

Cryo-electron microscopy (Cryo-EM) studies confirmed that the major species present in the sample was a higher-order oligomeric structure. The wild type protein could not be crystallized. In contrast to the wild type protein, each of the deletion constructs was monodisperse and adopted a single oligomeric state. The construct AgsA:DelC formed only 24-mers in solution. The only difference between AgsA:His-DD and AgsA:DD is the presence of an N-terminal tag on the former. However, in solution, they formed 24-mers and 18-mers, respectively (Fig. 3 and Table 1).

In the following sections we report the dimeric crystal structure of the core ACD of AgsA:His-DD at 2.0 Å resolution and low resolution (4.1 Å and 7.5 Å) oligomeric crystal structures of truncation constructs AgsA:DelC and AgsA:DD, respectively. AgsA:DD formed symmetric 18-mers, an oligomeric assembly not reported previously. AgsA:DelC, differing from AgsA:DD only in the N-terminus, formed 24-mers similar to those formed by *M. jannaschii* Hsp16.5 (MjHsp16.5). Cryo-EM studies of the wild type protein showed that the 18-meric oligomer of AgsA:DD could be easily fitted into the reconstructed envelope.

### Chaperone activity of all constructs

**Figure 4:**
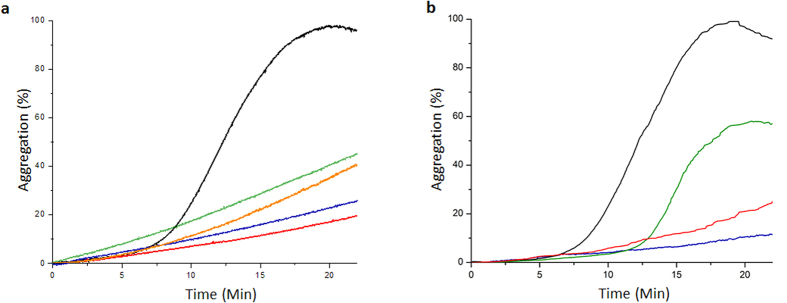
(**a**) Aggregation of 10 mM lysozyme (black), and 10 mM lysozyme in the presence of 10 mM of AgsA wild type (blue), AgsA:DD (green), AgsA:DelC (red) and AgsA:His-DD (orange). (**b**) Aggregation of 8 mM lysozyme (black), and 8 mM lysozyme in the presence of 8 mM of unseparated AgsA wild type (blue), separated 18-meric fraction of wild type (green) and separated 24-meric fraction of wild type (red).

All four constructs were able to prevent chemically denatured lysozyme from aggregating (Fig. 4a). Constructs possessing an intact N-terminal domain (AgsA wild type and AgsA:DelC) were better at preventing the aggregation of chemically denatured lysozyme. This result is consistent with previous studies on MjHsp16.5 and human αB-crystallin, which implicated the N-terminal domain in lysozyme recognition, binding and sequestration^11^,^12^. The two oligomeric fractions present in the wild type protein were separated and the chaperone activity of each fraction was measured. The activities of the separated populations showed considerable reduction compared to the wild type protein (Fig. 4b). These results, along with the SEC-MALS study clearly indicate that the 24-mers coexist with the 18-mers in dynamic equilibrium and this equilibrium is important for chaperone activity.

### Crystal structures

#### (i) AgsA:His-DD–a dimer

**Figure 5:**
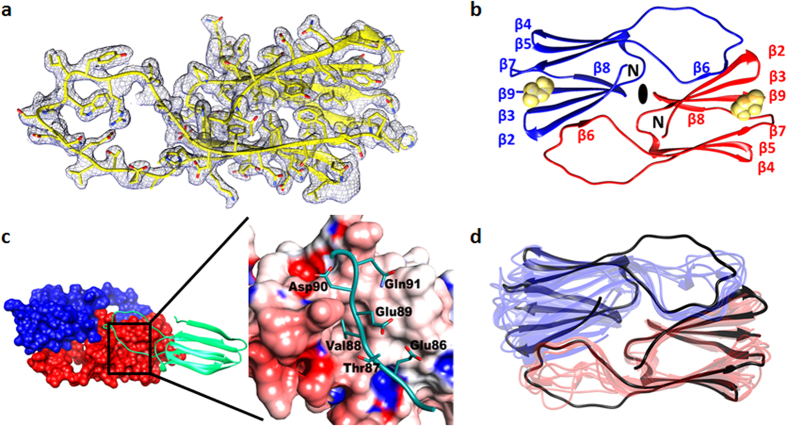
(**a**) Electron density of AgsA:His-DD monomer, contoured at 1.0 σ. (**b**) Structure of the symmetric ACD dimer. Protomers are in red and blue, Methyl Pentanediol molecule in yellow. (**c**) Binding of dimerization loop (green) in β4–β8 groove of neighbouring molecule in lattice (red and blue surface) (*Left*). Close-up of boxed region, loop shown in green sticks, groove as electrostatic surface (*Right*). (**d**) Superposition of AgsA:His-DD dimer (black) with all other non-metazoan sHSP dimers.

The core ACD fragment of AgsA:His-DD crystallized as a symmetric dimer in the space group *P*4_1_2_1_2 (Table 2). Mass spectrometry of the crystals revealed that the full length protein (size 17.1 kDa, 150 amino acids) had degraded in the crystallization condition to a fragment with mass 12.7 kDa, which corresponds to residues 35–147 (Figs 1 and 3c). Further truncations at the C-terminus account for the masses of other peaks in the mass spectrum. Continuous electron density could be traced for residues 40–132 (Fig. 5a), and the monomer adopts an α-crystallin-like fold, with residues 83–103 forming a long loop which separates two β-sheets. Residues 93–98 of this loop in subunit 1, form part of a β-sheet in subunit 2 (Fig. 5b). Terminal segments are required for the formation of higher oligomers of sHSPs and their truncation led to the formation of dimers.

The IXI motif in the C-terminal tail of sHSPs is known to mediate higher order oligomerization by binding in a hydrophobic cleft formed between strands β4 and β8 in an adjacent dimer. This inter-dimer interaction is observed between dimers in the oligomeric MjHsp16.5, *S. tokodaii* Hsp14, *S. pombe* Hsp16 and wheat Hsp16.9. In the dimeric structures of *D. radiodurans* Hsp17.7 and *S. tokodaii* Hsp14 double mutant^13^,^14^, similar interations are observed between symmetry related dimers. In the AgsA:His-DD dimer, this groove is occupied by the dimerization loop of a subunit belonging to a neighbouring dimer (Fig. 5c). This interaction buries 434 Å^2^ of surface area and involves five hydrogen bonds and one salt bridge. The hydrophobic cleft is known to be a substrate binding site^15^ and the binding of the eight residue stretch from the dimerization loop may represent the interaction between a sHSP and its substrate.

**Figure 6:**
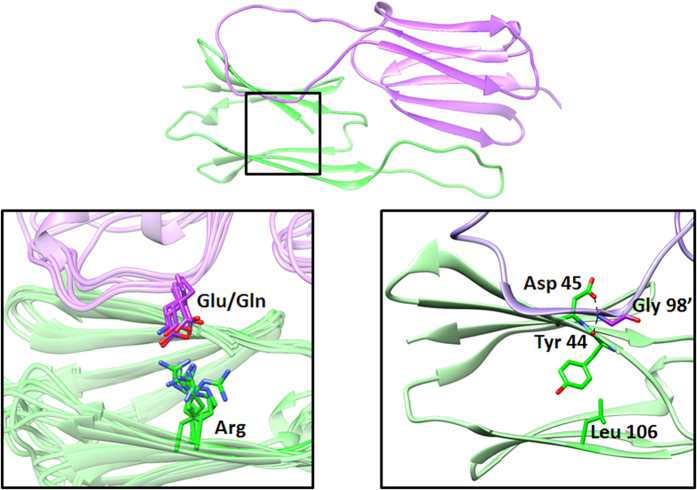
Inter-subunit salt bridge in sHSP dimers. Upper panel: Monomers are shown in green and lavender, box indicates region containing conserved arginine. Enlarged view of region in box is shown in lower panel. Left: Superposition of ACD dimers of *M.jannaschii* Hsp16.5 (PDB: 1SHS), wheat Hsp16.9 (PDB: 1GME), *S. pombe* Hsp16.0 (PDB: 3W1Z), *X. axonopodis* HspA (PDB: 3GLA), *S. tokodaii* Hsp14 (PDB: 1AAB), *D. radiodurans* Hsp17.7 (PDB: 4FEI). Right: Corresponding region in AgsA:HisDD dimer.

The dimerization loop of AgsA is wider than those of other sHSPs (Fig. 5d), while the buried surface area at the dimeric interface is the least. Unlike all other non-metazoan sHSP dimers which form 3–4 intra-dimer salt bridges, there are no salt bridges between the two subunits of the AgsA ACD dimer. A highly conserved feature of the dimerization loop in all non-metazoan sHSP dimers is the formation of an inter-subunit salt bridge between an arginine on the β7 strand and a glutamate in the loop. This strand participates in dimerization, stabilization of the β-sheet, and in chaperone function^16^. Mutations of this arginine in human sHSPs have been implicated in several diseases like cataracts, myopathies and axonal Charcot–Marie–Tooth disease^3^. Mutating the arginine to glycine in MjHsp16.5 and human αB-crystallin resulted in oligomers that were larger and more polydisperse than the wild type^17^. Structural and sequence alignments reveal that in AgsA, the equivalent residues to Arginine and Glutamate are Leu106 and Gly98, respectively (Fig. 1). Leu106 of subunit 1 makes no contact with the dimerization loop of subunit 2, whereas Gly98 of subunit 1 participates in the formation of two hydrogen bonds, one each with Tyr44 and Asp45 on the β2 strand of subunit 2 (Fig. 6).

#### (ii) AgsA:DD–an 18-mer

**Figure 7:**
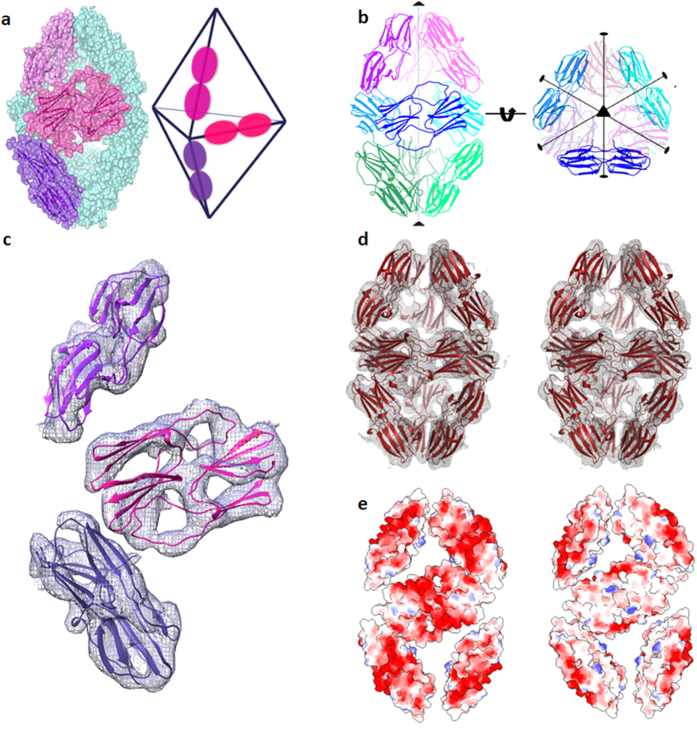
(**a**) 18-mer of AgsA:DD, ASU in shades of purple. (**b**) Symmetry elements of the 18-mer. (**c**) Electron density map of the ASU, contoured at 1.0 σ. (**d**) Stereo view of electron density map for the oligomer, contoured at 1.0 σ, obtained after molecular replacement. (**e**) Electrostatic surface representation of outside (left) and inside (right) of 18-mer.

AgsA:DD crystallized in the *R*32 space group with 3 dimers in the asymmetric unit (ASU) (Table 2). The application of the crystallographic 3-fold axis yields a closed 18-mer, with a dimer lying along each side of a trigonal bipyramid (Fig. 7a). The long axis of the oligomer that joins both the vertices is coincident with the crystallographic 3-fold axis. Three non-crystallographic 2-fold axes bisect each of the dimers that lie along the equatorial plane, perpendicular to the 3-fold axis (Fig. 7b). Thus, the 18-mer formed by nine dimers has a 32 symmetry. The only other 18-meric protein structure reported till date is a pore-forming bacterial toxin that adopts a doughnut-like arrangement of two nonameric rings^18^. Due to the low resolution of the data, initial molecular replacement (MR) attempts with monomers and dimers from all other known structures failed to yield a solution. In the meantime, we determined the structure of AgsA:His-DD dimer at 2 Å resolution, as described in the previous section. When this structure was used as a search model, an unambiguous MR solution of 3 dimers in the ASU was obtained, with clear and well-defined density for the β-sheets (Fig. 7c,d). The outer surface of the oligomer is charged, and its interior is hydrophobic (Fig. 7e). Long, continuous electron density is seen in the interior of the oligomer, which could account for the first 39 residues of the N-terminus. Though the IXI motifs in the C-termini of the 18-mer could not be defined for any monomer, the positioning of the monomers at inter-dimer interfaces indicates that the C-terminal regions are poised to form the hydrophobic interactions with the neighbouring β4–β8 clefts.

#### (iii) AgsA:DelC–a 24-mer

**Figure 8:**
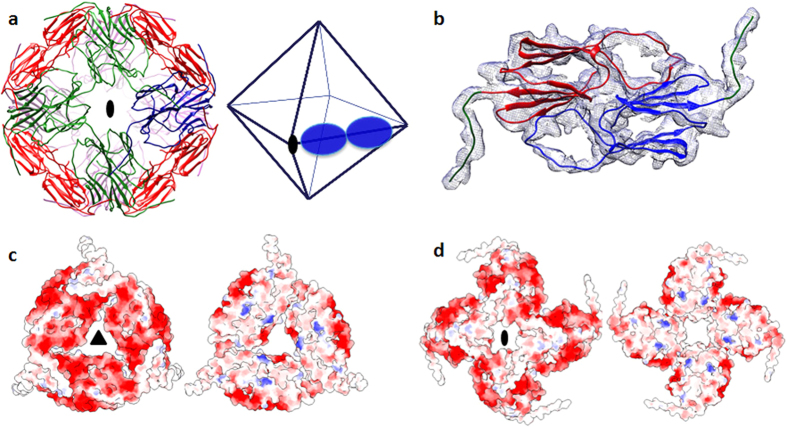
(**a**) 24-mer of AgsA:DelC, ASU shown in blue, crystallographic 2-fold axis is indicated. (**b**) Electron density map of ASU contoured at 1.0 σ. C-terminal extensions which were built during refinement are shown in green ribbon representation. Electrostatic surface representation of outside (left) and inside (right) of 24-mer viewed down (**c**) 3-fold axis, (**d**) 2-fold axis.

Cubic crystals of AgsA:DelC in the *I*23 space group diffracted to 4.1 Å resolution (Table 2). The application of crystal symmetry on the MR solution of a dimer in the ASU yielded an octahedral 24-mer, similar to the assembly of MjHsp16.5 and *S. tokodaii* Hsp14 (Fig. 8a). Though the search model lacked both termini for both protomers, successive rounds of refinement yielded clear density into which we could build the C-terminal extensions of both subunits (Fig. 8b). These termini containing the IXI motifs (residues 143–145: Ile-Ala-Ile), bind in the hydrophobic groove present between strands β4 and β8 of a neighbouring dimer related by a crystallographic 2-fold axis. As observed for MjHsp16.5 wild type and its N-terminal variant, this AgsA oligomer is also charged on the outside and hydrophobic inside^4^,^19^ (Fig. 8c,d).

### Cryo-EM reconstruction of AgsA wild type protein

**Figure 9:**
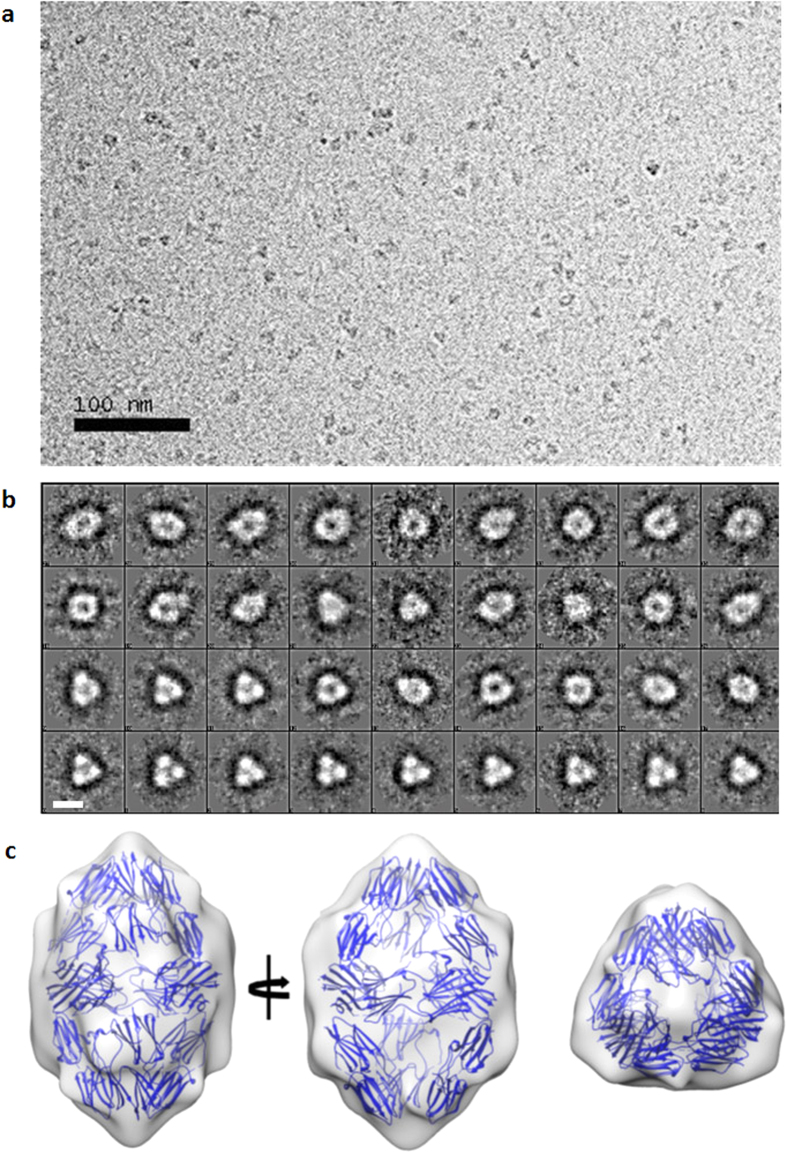
Cryo-EM studies of the wild type full-length AgsA. (**a**) Individual micrograph. (**b**) Class averages of the particles, white bar indicates scale (100 Å), and (**c**) Fit of the 18-mer of AgsA:DD crystal structure into the cryo-EM map; left and middle panels are views down the 2-fold axes, and right panel is view down the 3-fold axis.

Cryo-EM studies suggested that the major species present in the sample was a higher-order oligomeric structure with its shape and symmetry consistent with the crystal structure of AgsA:DD (Fig. 9a,b). The 18-meric oligomer of AgsA:DD could be easily fitted into the reconstructed envelope (Fig. 9c). These results are also in line with solution studies indicating that the wild type protein exists predominantly as an 18-mer. Together, these studies show that wild type AgsA very likely adopts the same 18-mer oligomeric configuration as observed in the crystal structure of the double deletion construct, AgsA:DD.

### Formation of different oligomeric states

**Figure 10:**
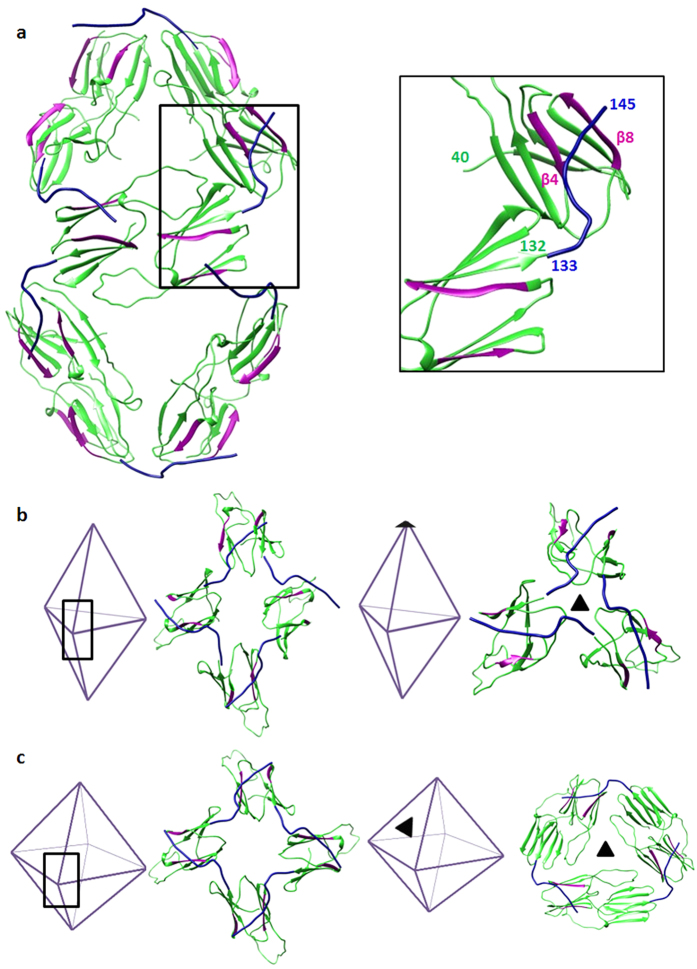
Superposition of C-terminal extension of AgsA:DelC on each monomer of AgsA:DD. (**a**) Residues 133–145 of AgsA:DelC (blue) occur in the vicinity of the β4–β8 groove (magenta) of AgsA:DD (green). The interface indicated by a box is enlarged on the right. Other subunits are omitted for clarity. (**b**) Superposition of C-terminal extensions of AgsA:DelC on monomers of AgsA:DD at different junctions in the 18-mer. Colour scheme same as that of Fig. 8a. (**c**) Interaction of C-terminal extension of AgsA:DelC (blue) with β4–β8 groove (magenta) of neighbouring dimers, at different junctions of 24-mer.

All non-metazoan ACD dimers display the same fold, but in spite of the conservation of their dimer structures, assemble into varied types of oligomers. A key difference between the reported oligomers lies in the hinge angle between the ACD and the C-terminal extension. The C-terminal extension in the AgsA:DelC construct forms crucial interactions to enable its assembly into a 24-mer. Upon superposition of the AgsA:DelC dimer on the 18-mer, C-terminal extensions are seen to occur in the vicinity of the β4–β8 groove (Fig. 10a). By varying the hinge angle, the C-terminal extensions of protomers in the 18-mer can bind in the hydrophobic grooves of neighbouring dimers. The network of such interactions, similar to those observed in wheat Hsp16.9 and *S. pombe* Hsp16 structures, mediate assembly of the closed 18-mer (Fig. 10b,c). sHSPs display remarkable heterogeneity in oligomer structure and here we demonstrate how an sHSP can adopt different quaternary structures via changes in the terminal regions.

## Discussion

Of late, there has been a paradigm shift in the understanding of sHSPs and their chaperone function. It is now accepted that sHSPs populate a range of oligomeric states at equilibrium, forming polydisperse and dynamic ensembles. Fluctuations in the terminal regions cause changes in inter-subunit contacts and this leads to a change in the distribution of various oligomeric species. Such changes in the population distribution are strongly correlated with sHSP chaperone activity and its regulation. However, the exact mechanism of this process, the contribution of different sequence and structural motifs to oligomeric interactions and chaperone function, and the cross-talk between the latter two properties are still poorly understood.

In line with the current understanding of sHSP structure and function, our results show that *in vivo*, the wild type protein exists in an equilibrium between two oligomeric states: 18-mer and 24-mer. Further, this equilibrium seems important for chaperone activity. The crystal structures we have described represent these oligomeric assemblies of the wild type protein.

AgsA:DD and AgsA:DelC crystallized as different oligomers: an 18-meric trigonal bipyramid and a 24-meric octahedron, respectively. AgsA:His-DD crystallized as a dimer following the loss of its terminal segments. Cryo-EM reconstructions indicate that the major species of wild type AgsA adopts the same 18-mer oligomeric configuration as AgsA:DD. The core structures of the dimers that constitute the different oligomers do not appear to show significant structural variations from the ACD dimer. This modular architecture of sHSPs facilitates the formation of different oligomers from the same building blocks. Our results support a model where multiple weak interactions promote oligomeric contacts^20^ and alterations in the equilibrium oligomer distribution can arise from fluctuations confined mainly to the terminal regions.

## Methods

### Cloning, over-expression and purification of AgsA wild type and truncation constructs

Genomic DNA of *Salmonella enterica* serovar *typhimurium* LT2 was obtained from BEI Resources, USA. Four constructs of AgsA were cloned into the pRSET C vector: 1) Wild type protein, tagless construct harbouring no affinity tags at either termini; 2) AgsA:DelC lacking 9 residues at the C-terminus, tagless; 3) Double deletion AgsA:DD lacking 11 residues at the N-terminus and 9 residues at the C-terminus, tagless and 4) AgsA:His-DD, a hexa-histidine tagged double deletion construct.

Clones were transformed into *E. coli* BL21 (DE3) and expression was induced by addition of 0.7 mM IPTG at 37 °C. Following centrifugation of the culture, cell pellets of the tagless constructs (Wild type protein, AgsA:DelC and AgsA:DD) were resuspended in buffer containing 20 mM bis-tris, 30 mM NaCl, 1 mM EDTA, 1 mM β-mercaptoethanol at pH 6.3. After cell lysis, the soluble cell fraction was purified by anion exchange chromatography through a Q-sepharose column. The protein was eluted with an NaCl gradient from 30–300 mM. Fractions containing the protein were identified by SDS-PAGE and passed through a GE fast flow Q-Sepharose column for a further round of purification. The eluted proteins were subjected to size exclusion chromatography (SEC) through a Sephacryl-500 column with buffer containing 20 mM Tris, 500 mM NaCl, 1 mM EDTA at pH 7.5. The protein was subjected to another round of purification by SEC through a Superose 6 10/300 column with buffer containing 20 mM Tris, 50 mM NaCl at pH 7.5. At all stages, we checked the purity of the protein by SDS-PAGE.

AgsA:His-DD construct was purified by immobilised metal affinity chromatography. The cell pellet was resuspended in buffer containing 20 mM tris, 200 mM NaCl, pH 7.5. Following cell lysis by sonication, the lysate was centrifuged and the supernatant was extracted and applied onto a Ni-NTA column pre-equilibrated with the lysis buffer. Protein was eluted from the column by passing lysis buffer supplemented with 200 mM imidazole. The elution fractions containing AgsA:His-DD were identified by SDS-PAGE and further purified by SEC through a Sephacryl-500 column.

### Solution studies

Dynamic light scattering experiments were performed on an Xtal concepts SpectroSize 300 instrument, with protein concentrations of 3–4 mg/ml. The hydrodynamic radius and polydispersity were determined using Spectrosize 300 software.

Analytical size exclusion chromatography was performed using a Superose 6 10/300 column with buffer containing 20 mM Tris, 50 mM NaCl at pH 7.5. For molecular weight estimation, calibration was performed using standard molecular weight markers: Ferritin, amylase, γ-globulin, ovalbumin and myoglobin.

For SEC-MALS, the buffer used was 20 mM Tris, 200 mM NaCl, pH 8.0 (for wild type protein) or 20 mM Tris, 50 mM NaCl, pH 7.5 (for separated fractions of wild type). The Superose 6 10/300 column (for wild type protein) or the Superdex 200 10/300 was connected to Wyatt Treos multi-angle light scattering instrument with an inline refractive index detector (Waters 2414 RI detector) and a multi-wavelength UV detector (Shimadzu SPD-10 A vp UV-Vis detector). Approximately 100 μL of 2 mg/mL protein solution was injected onto the column. The molar mass and mass percent of each fraction was calculated using the ASTRA program (Wyatt Technology).

### Chaperone activity assay

10 μM of lysozyme in 50 mM sodium phosphate buffer, pH 7.1, was denatured with 60 mM DTT at 37 °C. The aggregation of lysozyme was monitored for 20 minutes at 360 nm, in a JASCO double beam spectrophotometer. To assay chaperone activity of sHSPs, 10 μM of sHSP was added to the sample and reference cuvettes, prior to the addition of lysozyme in the sample cuvette. The chaperone activity of the separated fractions of the wild type protein were performed with 8 μM lysozyme denatured with 48 mM DTT. All other experimental conditions remained identical.

### Crystallization and X-Ray data collection

All crystallization trials were performed in a hanging drop vapour-diffusion set-up. 2.5 μl of protein (concentration of 7 mg/ml) was mixed with 2.5 μl of precipitant and equilibrated against a reservoir of 0.35 ml of precipitant, at a temperature of 20 °C. Crystals appeared in two weeks. The precipitants that yielded crystals for the various constructs were (i) AgsA:His-DD −25% Pentaerythritol Propoxylate (5/4 PO/OH), 0.1 M MES, pH 6.0, 20% MPD; (ii) AgsA:DD −30% Polypropylene glycol 400, 0.1 M NaCl; (iii) AgsA:DelC −30% Pentaerythritol Propoxylate (5/4 PO/OH), 0.1 M MES, pH 6.0 and 30% Glycerol.

Diffraction data for all crystals were collected at the BM14 beamline of ESRF, at a temperature of 100 K, at wavelengths of 0.97856 Å, 0.95372 Å and 0.97625 Å, respectively, for AgsA:His-DD, AgsA:DD and AgsA:DelC.

### Structure solution and refinement

For AgsA:His-DD, molecular replacement (MR) was performed with PHASER^21^, using the template of *S. pombe* Hsp16 (PDB ID: 3W1Z) without the N- and C-termini and dimerization loop. The solution had a TFZ of 4.4 and LLG 77. Refinement for AgsA:His-DD was completed through iterative rounds of manual model building in *Coot*^22^ and refinement using REFMAC5^23^. The final model consists of a continuous backbone for residues 40–132, of which 95.6% and 4.4% residues are in the favored and allowed regions of the Ramachandran plot, respectively^24^. The crystallization condition contained MPD, and one molecule of MPD was present in the ASU.

The dimer structure of AgsA:His-DD was used as the template for MR for all other data. For the low resolution data of AgsA:DD, initial MR trials with monomers, dimers, tetramers and hexamers of all other known structures failed to yield a solution. Only after we obtained the dimeric AgsA:HisDD structure, we were able to obtain an MR solution for AgsA:DD. A search for 3 copies of the AgsA:His-DD dimer with the program PHASER yielded a solution with clear electron densities for the chains and no clashes at the interface. For this solution, the TFZ was 5.2 and LLG was 95. A further search with this solution, i.e. combining the three dimers yielded a TFZ of 8.8 and LLG of 96. The MR solution was subjected to density modification and rebuilding through the programs RESOLVE^25^ and morph_model^26^ implemented in the PHENIX suite. This was followed by positional, rigid body and group B-factor refinement with the program phenix.refine^27^, while implementing NCS and reference structure (AgsA:His-DD) restraints.

For AgsA:DelC, MR placed an ACD dimer in the ASU with TFZ of 8.1 and LLG of 134. Though the model lacked the C-termini, successive rounds of refinement with phenix.refine yielded clear density for both C-terminal extensions, into which we were able to build 15 residues for each chain. NCS and secondary structure restraints were implemented through phenix.refine.

### Cryo-EM reconstruction

Cryo-EM experiments were performed on a JEM2010 (200 KV) cryo-electron microscope equipped with CCD camera at the National Center for Macromolecular Imaging, Baylor College of Medicine, USA. The wild type full-length AgsA was plunge-frozen using a Vitribot (MIV). The frozen specimen was imaged at 50000× magnification, with defocus levels ranging from −1.8 μm to −4.0 μm. The processing and preliminary three-dimensional reconstructions of the data were carried out using the protocols as implemented in the EMAN2 software package^28^. For the reconstructions of the wild type AgsA, ~5500 particle images (2.18 Å/pixel) were used, and D3 symmetry was enforced. The estimated resolution based on Fourier shell correlation was ~20 Å following iterative cycles of refinement starting from an initial model generated by EMAN2. The cryo-EM map was viewed with the molecular graphics software UCSF Chimera^29^. The oligomeric structures were fit into the reconstructed map using the ‘Fit in map’ utility of UCSF Chimera.

## Additional Information

**Accession Codes**: Coordinates and structural factors have been deposited in the RCSB Protein Data Bank. The PDB IDs are 4ZJ9 for AgsA:His-DD, 4ZJA for AgsA:DelC and 4ZJD for AgsA:DD.

**How to cite this article**: Mani, N. *et al*. Multiple oligomeric structures of a bacterial small heat shock protein. *Sci. Rep.* **6**, 24019; doi: 10.1038/srep24019 (2016).

## Figures and Tables

**Table 1:** Oligomeric states of different constructs of AgsA.

Construct	Oligomeric state in solution	Hydrodynamic diameter from dynamic light scattering (nm)	Oligomeric state in crystal structure	Dimensions from crystal structure (nm)
AgsA:DD	18	13.9	18	8.5 × 8.5 × 13.2
AgsA:DelC	24	11.4	24	11.2 × 11.2 × 11.2
AgsA:His-DD	24	10.2	2 (Residues 40–132)	

**Table 2:** Data collection and refinement statistics for all AgsA constructs.

	AgsA:His-DD	AgsA:DD	AgsA:DelC
Data collection			
Space group	*P*4_1_2_1_2	*R*32#	*I*23
Cell dimensions			
*a, b, c* (Å)	53.07, 53.07, 81.84	89.78, 89.78, 707.48	126.40, 126.40, 126.40
α, β, γ (°)	90, 90, 90	90, 90, 120	90, 90, 90
Resolution (Å)	34.12–2.00 (2.11–2.00)^*^	78.61–7.50 (7.91–7.50)	63.20–4.10 (4.32–4.10)
*R*_sym_ or *R*_merge_ (%)	6.4 (71.9)	8.9 (94.8)	8.0 (103.7)
*I*/σ*I*	22.9 (4.1)	8.0 (1.7)	14.8 (2.5)
Completeness (%)	99.8 (100)	100 (100)	100 (100)
Redundancy	13.8 (14.1)	4.9 (5.2)	10.6 (10.9)
Refinement			
Resolution (Å)	2.00	7.50	4.10
No. reflections	8413 (1189)	1625 (220)	2757 (387)
*R*_work_/*R*_free_ (%)	21.99/25.43	35.15/39.58	38.58/41.27
No. atoms			
Protein	717	4294	1599
Ligand/ion	8	0	0
Water	29	0	0
***B***-factors (Å^**2**^)			
Protein	58.79	333.0	187.36
Ligand/ion	73.11	−	−
Water	54.76	−	−
R.m.s. deviations			
Bond lengths (Å)	0.017	0.016	0.007
Bond angles (°)	1.991	2.918	1.539

^*^Values in parentheses are for highest-resolution shell.

^#^Data were processed in the hexagonal setting of space group *R*32.

## References

[b1] Haslbeck M. & Vierling E. A first line of stress defense: small heat shock proteins and their function in protein homeostasis. J. Mol. Biol. 427, 1537–1548 (2015). 25681016 10.1016/j.jmb.2015.02.002 PMC4360138

[b2] Arrigo A. Human small heat shock proteins: Protein interactomes of homo- and hetero-oligomeric complexes: An update. FEBS Lett. 587, 1959–1969 (2013). 23684648 10.1016/j.febslet.2013.05.011

[b3] Clark A. R., Naylor C. E., Bagnéris C., Keep N. H. & Slingsby E. Crystal structure of R120G disease mutant of human αb-crystallin domain dimer shows closure of a groove. J. Mol. Biol. 408, 118–134 (2011). 21329698 10.1016/j.jmb.2011.02.020 PMC3158665

[b4] Kim K. K., Kim R. & Kim S. H. Crystal structure of a small heat-shock protein. Nature 394, 595–599 (1998). 9707123 10.1038/29106

[b5] van Montfort R. L., Basha E., Friedrich K. L., Slingsby C. & Vierling E. Crystal structure and assembly of a eukaryotic small heat shock protein. Nat. Struct. Biol. 8, 1025–1030 (2001). 11702068 10.1038/nsb722

[b6] Hanazono Y. . Nonequivalence observed for the 16-meric structure of a Small Heat Shock Protein, SpHsp16.0, from *Schizosaccharomyces pombe*. Structure 21, 220–228 (2013). 23273429 10.1016/j.str.2012.11.015

[b7] Morrow G., Hightower L. E. & Tanguay R. M. Small heat shock proteins: big folding machines. Cell Stress Chaperones. 20, 207–212 (2015). 25536931 10.1007/s12192-014-0561-0 PMC4326388

[b8] Shi X. . Small heat shock protein AgsA forms dynamic fibrils. FEBS Lett. 585, 3396–3402 (2011). 22001209 10.1016/j.febslet.2011.09.042

[b9] Tomoyasu T. . A new heat shock gene, *AgsA*, which encodes a small chaperone involved in suppressing protein aggregation in *Salmonella enterica* serovar typhimurium. J. Bacteriol. 185, 6331–6339 (2003). 14563868 10.1128/JB.185.21.6331-6339.2003 PMC219406

[b10] Tomoyasu T., Tabata A. & Nagamune H. Investigation of the chaperone function of the small heat shock protein - AgsA. BMC Biochem. 11, 27 (2010). 20653971 10.1186/1471-2091-11-27 PMC2920228

[b11] Mainz A. . The chaperone αB-crystallin uses different interfaces to capture an amorphous and an amyloid client. Nat. Struct. Mol. Biol 22, 898–905 (2015). 26458046 10.1038/nsmb.3108

[b12] Shi J. . Cryoelectron microscopy analysis of small heat shock protein 16.5 (Hsp16.5) complexes with T4 lysozyme reveals the structural basis of multimode binding. J. Biol. Chem. 288, 4819–4830 (2013). 23277356 10.1074/jbc.M112.388132 PMC3576087

[b13] Bepperling A. . Alternative bacterial two-component small heat shock protein systems. Proc. Natl. Acad. Sci. USA 109, 20407–20412 (2012). 23184973 10.1073/pnas.1209565109 PMC3528540

[b14] Takeda K. . Dimer structure and conformational variability in the N-terminal region of an archaeal small heat shock protein, StHsp14.0. J. Struct. Biol. 174, 92–99 (2011). 21195185 10.1016/j.jsb.2010.12.006

[b15] Healy E. & King P. A mechanism of action for small heat shock proteins. Biochem. Biophys. Res. Commun. 417, 268–273 (2012). 22155251 10.1016/j.bbrc.2011.11.098

[b16] Siva Kumar L. V., Ramakrishna T. & Mohan Rao Ch. Structural and functional consequences of the mutation of a conserved arginine residue in αA and αB crystallins. J. Biol. Chem. 274, 24137–24141 (1999). 10446186 10.1074/jbc.274.34.24137

[b17] Quinlan R. A. . Changes in the quaternary structure and function of MjHSP16.5 attributable to deletion of the IXI motif and introduction of the substitution, R107G, in the α-crystallin domain. Phil. Trans. R. Soc. B 368, 1203–1227 (2013). 10.1098/rstb.2012.0327 PMC3638399 23530263

[b18] Leone P. . X-ray and cryo-electron microscopy structures of monalysin pore-forming toxin reveal multimerization of the pro-form. J. Biol. Chem. 290, 13191–13201 (2015). 25847242 10.1074/jbc.M115.646109 PMC4505573

[b19] Mchaourab H. S., Lin Y. & Spiller B. W. Crystal structure of an activated variant of small heat shock protein Hsp16.5. Biochemistry 51, 5105–5112 (2012). 22670769 10.1021/bi300525x PMC3384710

[b20] Hanozono Y., Takeda K., Yohda M. & Miki K. Structural studies on the oligomeric transition of a small heat shock protein, StHsp14.0. J. Mol. Biol. 422, 102–108 (2012). 10.1016/j.jmb.2012.05.017 22613762

[b21] McCoy A. J. . *Phaser* crystallographic software. J. Appl. Crystallogr. 40, 658–674 (2007). 19461840 10.1107/S0021889807021206 PMC2483472

[b22] Emsley P., Lohkamp B., Scott W. G. & Cowtan K. Features and development of *Coot*. Acta Crystallogr. D 66, 486–501 (2010). 20383002 10.1107/S0907444910007493 PMC2852313

[b23] Murshudov G. N., Vagin A. A. & Dodson E. J. Refinement of macromolecular structures by the maximum-likelihood method. Acta Crystallogr. D 53, 240–255 (1997). 15299926 10.1107/S0907444996012255

[b24] Chen V. B. . MolProbity: all-atom structure validation for macromolecular crystallography. Acta Crystallogr. D 66, 12–21 (2010). 20057044 10.1107/S0907444909042073 PMC2803126

[b25] Terwilliger T. C. Automated main-chain model building by template matching and iterative fragment extension Acta Crystallogr. D 59, 38–44 (2003). 12499537 10.1107/S0907444902018036 PMC2745878

[b26] Terwilliger T. C. . Improved crystallographic models through iterated local density-guided model deformation and reciprocal-space refinement. Acta Crystallogr. D 68, 861–870 (2012). 22751672 10.1107/S0907444912015636 PMC3388814

[b27] Afonine P. V. . Towards automated crystallographic structure refinement with phenix.refine. Acta Crystallogr. D 68, 352–367 (2012). 22505256 10.1107/S0907444912001308 PMC3322595

[b28] Tang G. . EMAN2: an extensible image processing suite for electron microscopy. J. Struct. Biol. 157, 38–46 (2007). 16859925 10.1016/j.jsb.2006.05.009

[b29] Pettersen E. F. . UCSF Chimera-a visualization system for exploratory research and analysis. J Comput Chem. 25, 1605–1612 (2004). 15264254 10.1002/jcc.20084

